# HDL-apoA-I Exchange: Rapid Detection and Association with Atherosclerosis

**DOI:** 10.1371/journal.pone.0071541

**Published:** 2013-08-28

**Authors:** Mark S. Borja, Lei Zhao, Bradley Hammerson, Chongren Tang, Richard Yang, Nancy Carson, Gayani Fernando, Xiaoqin Liu, Madhu S. Budamagunta, Jacques Genest, Gregory C. Shearer, Franck Duclos, Michael N. Oda

**Affiliations:** 1 Children's Hospital Oakland Research Institute, Oakland, California, United States of America; 2 Cardiovascular Drug Discovery, Bristol-Myers Squibb Company, Pennington, New Jersey, United States of America; 3 Accent Assays, Inc., Sacramento, California, United States of America; 4 Department of Medicine, University of Washington, Seattle, Washington, United States of America; 5 Department of Biochemistry and Molecular Medicine, University of California Davis, Davis, California, United States of America; 6 Royal Victoria Hospital, McGill University Health Centre, Montreal, Quebec, Canada; 7 Cardiovascular Health Research Center, Sanford Research/USD, Sioux Falls, South Dakota, United States of America; 8 Department of Internal Medicine and Department of Basic Biomedical Sciences, Sanford School of Medicine, University of South Dakota, Sioux Falls, South Dakota, United States of America; Harvard Medical School, United States of America

## Abstract

High density lipoprotein (HDL) cholesterol levels are associated with decreased risk of cardiovascular disease, but not all HDL are functionally equivalent. A primary determinant of HDL functional status is the conformational adaptability of its main protein component, apoA-I, an exchangeable apolipoprotein. Chemical modification of apoA-I, as may occur under conditions of inflammation or diabetes, can severely impair HDL function and is associated with the presence of cardiovascular disease. Chemical modification of apoA-I also impairs its ability to exchange on and off HDL, a critical process in reverse cholesterol transport. In this study, we developed a method using electron paramagnetic resonance spectroscopy (EPR) to quantify HDL-apoA-I exchange. Using this approach, we measured the degree of HDL-apoA-I exchange for HDL isolated from rabbits fed a high fat, high cholesterol diet, as well as human subjects with acute coronary syndrome and metabolic syndrome. We observed that HDL-apoA-I exchange was markedly reduced when atherosclerosis was present, or when the subject carries at least one risk factor of cardiovascular disease. These results show that HDL-apoA-I exchange is a clinically relevant measure of HDL function pertinent to cardiovascular disease.

## Introduction

Plasma levels of high density lipoprotein cholesterol (HDL-C) are associated with a lower risk of cardiovascular disease [1]–[3]. This has been ascribed to HDL's atheroprotective properties, which include mediation of reverse cholesterol transport (RCT), down-regulation of inflammation, maintenance of endothelial function, and modulation of immune response [4]. Yet not all HDL are functionally equivalent as demonstrated by the relationship between HDL dysfunction and inflammation [4], [5]. In humans undergoing an acute phase response, HDL are pro-inflammatory [6], while HDL from type 2 diabetics exhibit lower endothelial protective properties [7]. These changes are likely the result of extensive HDL remodeling, as observed during induction of inflammation in mice, or in humans during acute phase response, resulting in impaired RCT [8]–[10].

The functional status of HDL is closely linked to its primary protein component, apolipoprotein A-I (apoA-I) [5], an abundant, multifunctional exchangeable apolipoprotein whose plasma concentration is inversely correlated with the incidence of coronary artery disease (CAD). This relationship is dependent on apoA-I's ability to mediate the interaction between HDL and key cholesterol transporters in the RCT pathway: ATP binding cassette transporter A1 (ABCA1) and scavenger receptor B1 (SR-B1) [11], as well as HDL remodeling factors such as lecithin:cholesterol acetyl transferase (LCAT) [12], [13], cholesterol ester transfer protein (CETP) [14], phospholipid transfer protein (PLTP) [15], [16], and endothelial lipase (EL) [17], [18].

ABCA1's primary substrate is lipid free/lipid-poor apoA-I [19]–[22]. In the arterial wall, desorption of lipid free/lipid-poor apoA-I from HDL is the most plausible source of cholesterol acceptor required to initiate de novo ABCA1-mediated cholesterol efflux and is the rate-limiting factor in cholesterol mobilization [23], [24]. Experiments conducted in our laboratory demonstrate that lipid-poor apoA-I is displaced from HDL by the binding of exogenous apolipoproteins, and chemical modifications that reduce HDL's steroid efflux capacity also impair HDL's ability to release lipid-poor apoA-I [25]. As a result, we hypothesize that apoA-I's displacement/desorption rate from HDL provides an indirect measure of HDL's cholesterol efflux capacity.

HDL dysfunction may arise from diabetes and/or oxidation-induced posttranslational modification of apoA-I, and is associated with atherosclerosis and progression of cardiovascular disease [4], [5], [26]. Non-enzymatic glycation of apoA-I in type 2 diabetics is implicated in the progression of coronary artery plaque formation, decreased LCAT activity, and impaired anti-inflammatory properties [27], [28]. Modification of specific lysine residues on apoA-I by reactive carbonyls such as malondialdehyde and acrolein blocks ABCA1-mediated cholesterol efflux [29], [30]. The oxidative enzyme myeloperoxidase (MPO) is expressed in macrophages and protects against invading pathogens by producing reactive oxygen species, and importantly, is also a leading cause of apoA-I modification [31]–[36]. MPO-modified apoA-I is localized to atherosclerotic lesions and is detectable in the plasma of coronary artery disease patients [34], [37]–[42]. Site-specific chlorination of tyrosine disrupts ABCA1-mediated cholesterol efflux [43], while sulfoxidation of methionine impairs both cholesterol efflux and LCAT activation [44], [45]. MPO oxidation also reduces the ability of apoA-I to exchange between lipid-associated and lipid-free states, a property of apoA-I that is critical for ABCA1-mediated cholesterol efflux [25]. These studies demonstrate that apoA-I is exquisitely sensitive to chemical modifications, which have profound effects on HDL function.

The conformational plasticity of apoA-I governs its ability to form HDL and undergo remodeling, thus measuring apoA-I's conformational adaptability may shed light on an aspect of HDL function highly relevant to CAD. To investigate this relationship, we developed a fluorescence-based approach to measure the rate of HDL-apoA-I exchange (HAE) in a reconstituted system [25]. Our findings suggested that HDL function was highly dependent on the conformational adaptability of apoA-I. However, the inherent fluorescence of blood plasma and serum limits the clinical utility of this approach to quantifying HAE.

We hypothesized that HAE is a key metric of the functional status of HDL, and that loss of this function is associated with a reduced ability of HDL to prevent or reverse atherosclerosis. Here, we describe a new method that employs site-directed spin-label electron paramagnetic resonance (SDSL-EPR, [46], [47]) to measure HAE of HDL in plasma and purified HDL. By utilizing SDSL-EPR, we take advantage of the absence of an EPR background signal in blood plasma and serum. In a rabbit model of atherosclerosis, we found that loss of HAE correlates with increasing atherosclerotic plaque burden. Furthermore, we demonstrate that HDL from patients with acute coronary syndrome (ACS) and metabolic syndrome are dramatically less capable of performing HAE, suggesting that apoA-I exchangeability is an important aspect of HDL function in cardiovascular disease.

## Materials and Methods

### Ethics Statement

Human study protocols were approved by the Research Ethics Board of McGill University Health Center Research Institute (MUHC-RI), the Institutional Review Board of Children's Hospital Oakland Research Institute (CHORI), the Institutional Review Board of the University of South Dakota, and Bristol-Myers Squibb. Written informed consent was obtained from all study participants. Animals were cared and subjected to procedures according to the *Guide for the Care and Use of Laboratory Animals* as approved by the Institutional Animal Care and Use Committee of Bristol-Myers Squibb.

### Human Plasma Samples

Blood samples from ACS subjects (n = 16) were collected 3 months following clinical presentation in the ER. The diagnosis of ACS was confirmed by clinical assessment by a cardiologist (JG), including ECG changes, troponin I elevation, and the presence of CAD on coronary angiography. Exclusion criteria included uncontrolled hypertension, triglycerides ≥443 mg/dL, severe obesity (BMI≥30), alcohol intake >14 drinks/week, and the presence of thyroid, hepatic or renal disease. Additionally, subjects were excluded if they had an autoimmune disease or any chronic or acute infectious or inflammatory illness. The protocol for blood sampling for this study was approved by the Research Ethics Board of MUHC-RI. All subjects provided informed consent to participate in this study. For the control arm of the study, plasma was collected from healthy, fasted donors (n = 9) who provided written informed consent for a study protocol approved by the Institutional Review Board of Children's Hospital Oakland Research Institute. Exclusion criteria included family history of coronary artery disease and hypertension.

Metabolic syndrome subjects were recruited and assessed as described [48]. Exclusion criteria included presence of cardiovascular disease and/or diabetes mellitus. Informed consent was obtained prior to the study and the protocol was approved by the Institutional Review Board at the University of South Dakota. The study was registered at clinicaltrials.gov (NCT00286234).

For all blood samples, plasma was isolated within 1 hour of collection and samples were stored at −70 to −80°C.

### Plasma Lipids

Plasma lipid and lipoprotein levels were measured by standard clinical laboratory techniques. All subjects were fasted for at least 8 hours prior to blood collection and lipid panel analyses.

### Native Non-denaturing Gradient Gel Electrophoresis

Alexa350-labeled apoA-I [49] was added in increasing concentrations with human plasma and incubated for 2 hours at 37°C. Gel electrophoresis was performed as described [50]. Fluorescence imaging was performed using an Alpha Imager employing an Alexa350 filter set.

### Production of Recombinant Spin-Labeled apoA-I Protein

Human apoA-I was expressed in *E. coli* using PET-20b (Novagen, Inc.) as described [50]. Expressed protein was purified using Hi-Trap nickel affinity columns (GE Healthcare) and nitroxide spin-labeled with methanethiosulfonate (MTS) spin-label as described [47], [51]. Briefly, protein was incubated with 100 µM TCEP and 300 µM MTS in a Hi-Trap nickel affinity column in the presence of 3 M guanidine HCL, followed by extensive washing in high-salt PBS (20 mM phosphate, 500 mM NaCl, pH 7.4) and elution with 500 mM imidazole in PBS. Protein purity (95%) was assessed by SDS-PAGE.

### Oxidation of HDL by MPO

Whole blood was drawn from Bristol-Myers Squibb employee volunteers (n = 3) who gave written, informed consent (RD-DIR 003) for research purposes in accordance with the Corporate Policy on Environment, Health and Safety (BMS-CP-004) and Corporate Health and Fitness (BMS-CP-035). HDL was isolated from human plasma (d = 1.063–2.210 g/mL) using differential ultracentrifugation and dialyzed in 50 mM phosphate buffer, pH 7.0 with 100 µM diethylenetriamine pentaacetic acid (DTPA) and 100 µM butylated hydroxytoluene (BHT) at 4°C. MPO oxidation of HDL was performed based on a modified protocol from Zheng, et al [52]. Briefly, MPO-mediated HDL oxidation was carried out in PBS (10 mM phosphate pH 7.4, 150 mM NaCl) containing 100 µM DTPA, 10 mg/dL apoA-I, and 50 nM purified human MPO (Calbiochem). Reactions were initiated by adding H_2_O_2_ at varying concentrations (0–300 µM). After 30 minutes incubation at 37°C, the reaction was terminated by adding 2 mM methionine and 0.3 µM catalase. The modified HDL was kept at 4°C until apoA-I exchange was evaluated by EPR.

### ABCA1 efflux capacity of serum HDL

Serum was derived from plasma by adding calcium. Polyethylene glycol (PEG) was then used to precipitate lipoproteins containing apolipoprotein B, and the supernatant was centrifuged to generate serum HDL [53], [54]. ABCA1-specific sterol efflux to serum HDL was quantified using baby hamster kidney (BHK) cells expressing mifepristone-inducible human ABCA1. BHK cells were radiolabeled with [^3^H]cholesterol for 24 h. Expression of ABCA1 was induced by incubating the cells for 20 h with DMEM containing 1 mg/mL bovine serum albumin (DMEM/BSA) and 10 nM mifepristone. Efflux of [^3^H]cholesterol was measured after a 4 h incubation with DMEM/BSA without or with 2% (v:v) serum HDL or 30 µg/ml ultracentrifugation isolated HDL. Cholesterol efflux mediated by HDL was calculated as the percentage of total [^3^H]cholesterol (medium plus cell) released into the medium after the value obtained with DMEM/BSA alone was subtracted [55].

### Rabbit Atherosclerosis Model

#### Animals and Diet

New Zealand White rabbits (n = 8) weighing 2.5–3.0 kg (Harlan Laboratories) were singly housed, cared and subjected to procedures according to the *Guide for the Care and Use of Laboratory Animals* as approved by the Institutional Animal Care and Use Committee of Bristol-Myers Squibb (Hopewell, NJ). Upon acclimation, animals were fed with normal rabbit chow diet (Teklad 203), then switched to pro-atherogenic diet Teklad C30255 (0.3% cholesterol, 4.7% coconut oil) for 3 months. Rabbits were bled via central ear artery using a catheter at 0, 2 and 3 months. Animals were anesthetized and sacrificed at 3 months.

#### Rabbit Plasma Lipid Analysis

EDTA-anti-coagulated blood samples were taken following an overnight fast and plasma was isolated by centrifugation. Plasma total cholesterol and triglyceride levels were determined enzymatically using Olympus AU680 Chemistry Analyzer.

#### HDL Isolation

HDL was prepared from EDTA-anti-coagulated blood of chow and high fat diet fed rabbits. HDL (d = 1.063–1.210 g/mL) was isolated by a sequential density gradient ultracentrifugation method adapted from Hoofnagle, et al [56]. Plasma was brought to a density of 1.21 g/mL with solid KBr, loaded into an ultracentrifuge tube, and gently overlaid with normal saline adjusted with KBr to a density of 1.21 g/mL. After ultracentrifugation at 70,000× *g* for 16 h, lipoproteins in the top 20% of the tube were collected, the density of the solution was adjusted to 1.063 g/mL, and the samples were centrifuged at 70,000× *g* for 16 h. Isolated HDL was dialyzed for 6 hours in phosphate buffer supplemented with 100 µM butylated hydroxytoluene, and 100 µM diethylenetriaminepentaacetic acid. Protein concentration was determined by the Bradford assay (Pierce) and lipid profile was determined using an Olympus AU680 chemistry analyzer (Olympus America, Inc.). Lipoproteins were stored at 4°C prior to analysis.

#### Histology Assessment of Aortic Atherosclerosis

Upon sacrifice, rabbit aortas were dissected and divided into 9 segments. Tissue segments were fixed in 10% Neutral buffered Formalin (Sigma Aldrich) and then prepared for histological analysis using a Leica ASP300S Tissue processor, employing 10% Neutral Buffered Formalin, with ascending concentrations of alcohol and xylene at ambient temperature and then paraffin (Paraplast X-TRA tissue embedding medium, Leica) at 60°C. Plaque burden was quantified by histological analysis of lesions (n≥3 per segment) using 6 µm thick serial paraffin sections stained with trichrome and counterstained with Hematoxylin, Gills Formula (Vector Labs), dehydrated and cleared with xylene. Atherosclerotic lesion area was measured using an in-house segment imaging software.

#### Rabbit RCT method

Cholesterol efflux activity assays were performed using protocol adapted from Asztalos, et al [57]. Briefly, J774 cells were plated in 96-well plates, cultured in RPMI media (Gibco) with 10% FBS. On day 2, cells were loaded with 2 µCi/mL ^3^H-cholesterol (Perkin Elmer) in the presence of 2 µg/mL ACAT inhibitor DUP-128 in assay media with 1% FBS for 24 hrs. To upregulate ABCA1, cells were treated with 8-CPT (Sigma) in complete media lacking FBS plus 0.2% FA-free albumin (Sigma) for an additional 18 hrs. On day 4, for cholesterol efflux measurements, cells were washed, and incubated with 2.8% rabbit APO-B depleted serum, along with apoA-I and human serum positive controls, and no serum (negative control) in 100 µl of complete medium lacking FBS plus 0.2% BSA. The following day culture media and isopropyl/heptane cell extract were transferred to a 96-well Optiplate and combined with microscint for determination of ^3^H-cholesterol using a microplate scintillation counter (Perkin Elmer, model C991200). Cholesterol efflux was calculated as the amount of serum-dependent radioactivity present in the media as a percentage of total [^3^H]-cholesterol in media plus cells.

### HDL-ApoA-I Exchange Assay

#### Sample Preparation

Freshly thawed plasma was mixed 1∶4 with PBS (20 mM phosphate, 150 mM NaCl, pH 7.4) and 24% w/v PEG 6000 (Sigma) was added to a final concentration of 4%. Samples were centrifuged at 13,000 rpm for 10 minutes in a tabletop centrifuge at 4°C to remove apoB-containing lipoproteins. The clarified plasma was then mixed with 3 mg/mL spin-labeled apoA-I in a 4∶1 ratio and drawn into an EPR-compatible borosilicate capillary tube (VWR). For purified HDL samples, spin-labeled apoA-I was added directly in a 4∶1 sample∶apoA-I ratio without further clarification.

#### EPR Spectroscopy

EPR measurements were performed with a Bruker eScan EPR spectrometer outfitted with temperature controller (Noxygen). Samples were scanned first at 6°C, incubated for 15 minutes at 37°C, and scanned again at 37°. The peak amplitude of the nitroxide signal from spin-labeled apoA-I in the sample (3462–3470 Gauss) was compared to the peak amplitude of a proprietary internal standard (3507–3515 Gauss) provided by Bruker. The internal standard is contained within the eScan spectrometer cavity and does not contact the sample. Since the y-axis of an EPR spectrum is measured in arbitrary units, measuring the sample against a fixed internal standard facilitates normalization of sample response. HAE activity was determined by subtracting the sample∶internal standard ratio obtained at 6°C from the sample∶internal standard ratio at 37°C. The baseline spectra of spin-labeled apoA-I in PBS was subtracted from results. Maximum amplitude of spin-labeled apoA-I was determined from spin-labeled apoA-I in an extended lipid-bound conformation. All samples were read in triplicate and averaged. Response of spin-labeled apoA-I in plasma or purified HDL was calculated as follows:

R_sample_ = (37°C peak amplitude)—(6°C peak amplitude) of spin-labeled apoA-I in plasma or HDL sample.

R_min_ = (37°C peak amplitude)—(6°C peak amplitude) of spin-labeled apoA-I in PBS.

R_max_ = (37°C peak amplitude)—(6°C peak amplitude) of spin-labeled apoA-I in PBS+SDS.
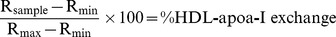



#### Statistical Analysis

Results are reported as mean ± SD. Differences between means were determined by performing unpaired, 2-tailed Student's *t*-test, one-way ANOVA, or two-way ANOVA with post hoc comparisons by custom tests of parameters. For two-way ANOVA, interaction p-values greater than 0.20 were dropped in favor of pooled estimates. Associations between different parameters were established by linear regression. All statistical analyses were performed using GraphPad Prism 5 or JMP software 9.0.3 (SAS Institute, Cary, NC). Statistical significance was assumed for *P*<0.05.

## Results

### ApoA-I is Specific for HDL

**Figure 1:**
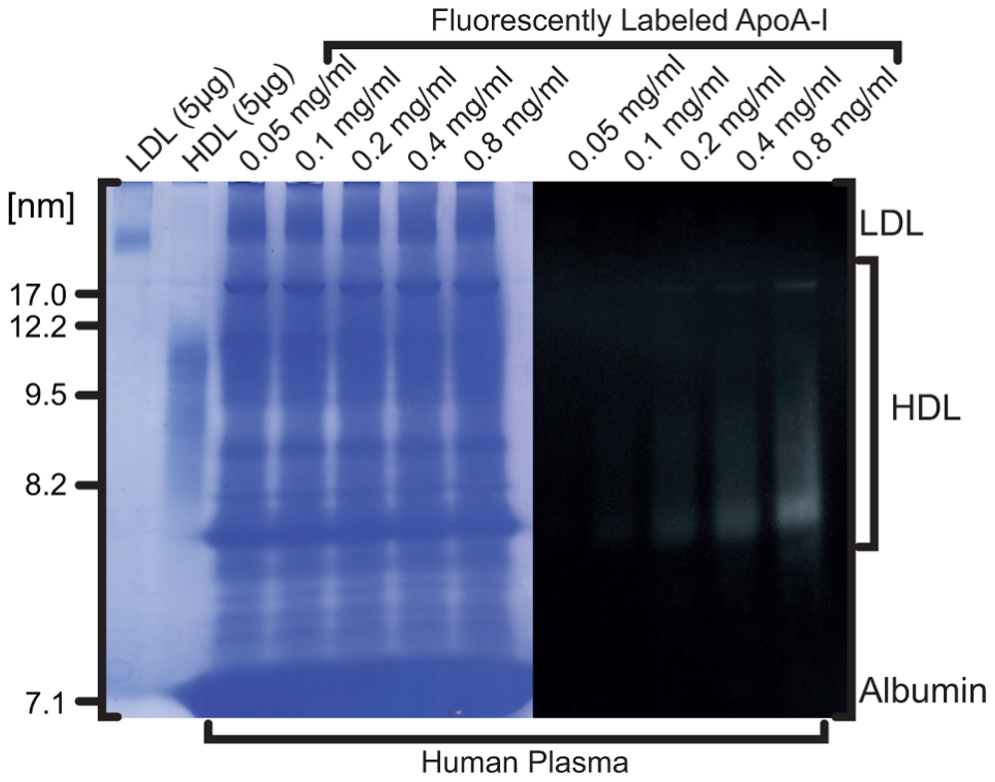
Non-denaturing gradient gel of fluorescently-labeled apoA-I added to human plasma. ApoA-I labeled with Alexa350 was incubated at 37°C at increasing concentrations with plasma from a healthy human donor and examined by polyacrylamide gel electrophoresis on a 4–20% Tris-glycine gel. Lanes 1 and 2 contain reference samples of purified LDL and HDL, respectively. Fluorescence imaging was performed prior to staining with Coomassie (excitation light, 365 nm; emission blue filter 440–480 nm). The figure is a composite of fluorescence and protein stain images of the same gel.

ApoA-I freely exchanges with HDL in a reconstituted system [25], and in human plasma. We verified apoA-I's specificity for HDL in human plasma, as has been observed in other laboratories that employed radioactive apoA-I [58]. To determine the specificity of labeled lipid-free apoA-I for HDL, recombinant single cysteine substituted apoA-I was labeled with Alexa350 fluorophore and incubated with human plasma for 2 hours at 37°C. The lipoprotein association of exogenous apoA-I was determined by native non-denaturing gradient gel electrophoresis. Fluorescence imaging revealed that Alexa350 apoA-I associated primarily with a particle of 7.8–8.0 nm in diameter, and to a minor extent with larger HDL particles (Figure 1). Exogenously added apoA-I did not bind to the VLDL, LDL or albumin regions, demonstrating that recombinant apoA-I remains highly specific to HDL. Furthermore, HDL-apoA-I exchange (HAE) occurs at physiological concentrations, and within a 2-hour timeframe.

### EPR-based Measurement of HDL-ApoA-I Exchange

Similar to the fluorescence method for quantifying apoA-I exchange with reconstituted HDL [25], the rate of HAE in blood plasma and with purified HDL was determined by EPR spectroscopy. EPR was employed because of the highly variable inherent fluorescence of blood plasma and serum. Because EPR monitors the spin resonance of free radicals, there is no background signal in either blood plasma or serum. EPR can detect conformational changes in nitroxide-labeled apoA-I and we have utilized this methodology to examine the structure of apoA-I on lipid-free and lipid-bound apoA-I [47], [49], [59]–[61]. Upon HDL-binding, apoA-I undergoes a significant conformational change, which is observed as an increase in nitroxide signal amplitude as apoA-I transitions from lipid-free to lipid-bound states (for examples, see ref. [47]).

**Figure 2:**
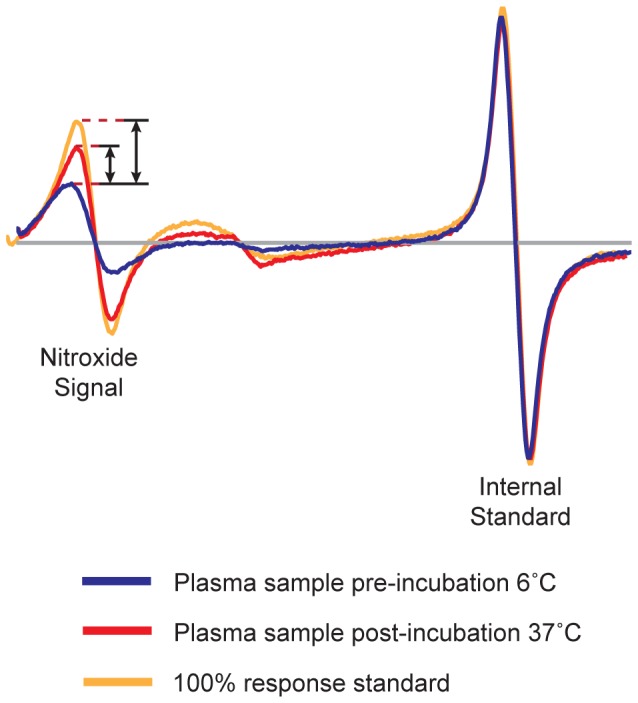
Representative EPR spectra of spin-labeled apoA-I probe added to human plasma. The spectrum of spin-labeled apoA-I in apoB depleted plasma from a healthy human donor at 6°C (blue line) is compared to the spectra after a 15 minute incubation at 37°C (red line). The maximal nitroxide spectral response is obtained from spin-labeled apoA-I in an extended lipid-bound conformation (orange line). Sample response was normalized between instruments using a proprietary internal standard. Sample response was calculated by subtracting the peak amplitude at 6°C from the amplitude at 37°C and dividing by the amplitude of the 100% response standard.

HAE was quantified by comparing the EPR peak amplitude of spin-labeled apoA-I in plasma at 6°C to that of spin-labeled apoA-I in plasma after a 15-minute incubation at 37°C (Figure 2). No further increases in nitroxide signal amplitude were observed when sample EPR spectra were monitored an additional 6 hours (data not shown), indicating that equilibrium between exogenous apoA-I and blood plasma HDL is achieved within 15 minutes. Additionally, we have determined that the rate and endpoint of HAE are highly correlated (data not shown). For the purposes of this manuscript, all HAE data were collected by endpoint analysis.

### Oxidation of HDL by MPO Impairs HDL-ApoA-I Exchange

**Figure 3:**
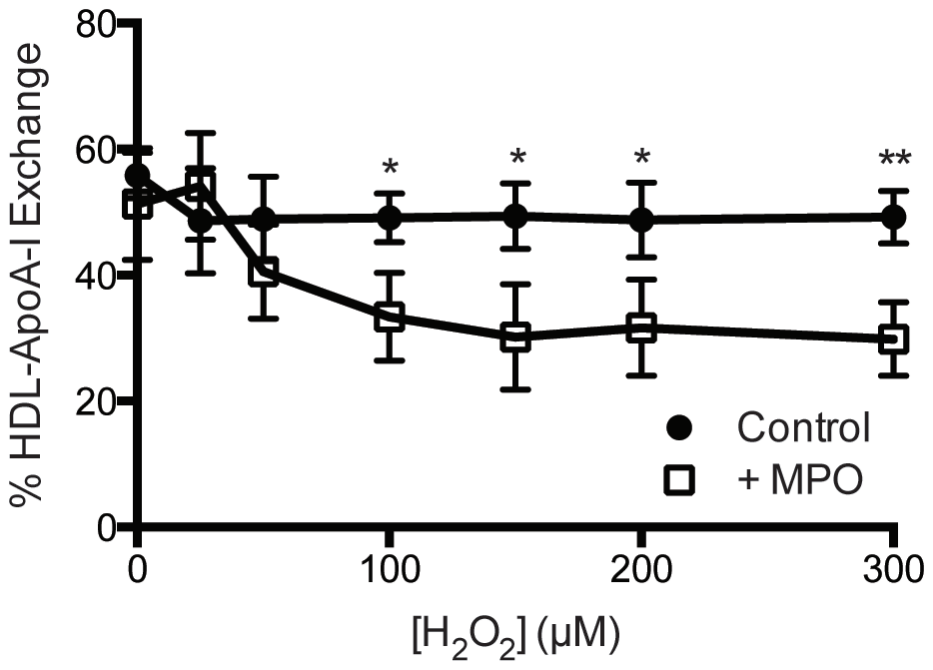
Oxidation of HDL by myeloperoxidase inhibits HDL-ApoA-I exchange. Human HDL from healthy, fasted volunteers (n = 3) was incubated at 37°C with increasing concentrations of H_2_O_2_ in the presence (open squares) or absence (closed circles) of 50 nM MPO. Reactions were performed at a constant apoA-I concentration of 10 mg/dL. Following oxidation, HAE was analyzed by EPR as described in materials and methods. Statistically significant differences are indicated (*P<0.05 and **P<0.01). Two-tailed Student's *t*-tests were performed on each pair of samples at their respective concentration.

Previously, we observed that oxidation of lipid-free apoA-I by MPO reduces the rate of HAE with reconstituted HDL [25], concomitant with diminished cholesterol efflux capacity [44], [45]. To determine if impairment of HAE by MPO oxidation could be detected by EPR, we exposed purified human HDL to MPO-H_2_O_2_ or H_2_O_2_ alone for 30 minutes at 37°C. ApoA-I concentration was kept constant at 10 mg/dL across samples. Concentrations of MPO and H_2_O_2_ were in the physiological to pathological range [52], and HAE was measured by EPR, as described above. While samples incubated with H_2_O_2_ alone showed only minor loss of HAE, samples exposed to the MPO-H_2_O_2_ system had significant declines in HAE in a dose-dependent manner (Figure 3), consistent with results from the fluorescence-based HAE assay [25], and with ABCA1-dependent cholesterol efflux in previous studies [53], [62]. Thus, the EPR-based approach is sufficiently sensitive to detect chemically-induced changes in HDL with physiologically relevant concentrations of MPO and H_2_O_2_.

### Atherosclerosis is Associated with Reduced HDL-ApoA-I Exchange and Cholesterol Efflux Capacity in Rabbits

To determine the relationship between HAE and atherosclerosis, we examined the effect of a high fat, high cholesterol diet on HAE in a rabbit model of atherosclerosis. Rabbits (n = 8) were fed normal rabbit chow and then switched to a high fat, high cholesterol diet containing 0.3% cholesterol for 12 weeks. Plasma was collected at 0, 8 and 12 weeks and lipid profiles verified hypercholesterolemia with TC ∼1100 mg/dl at 1 month and ≥1600 mg/dl at 3 months in these rabbits. At 12 weeks the animals were sacrificed and total plaque area from the aortic arch to the abdominal region was determined using histology analysis. Since rabbits have significantly less circulating HDL than humans, for HAE analysis HDL was isolated by ultracentrifugation.

**Figure 4:**
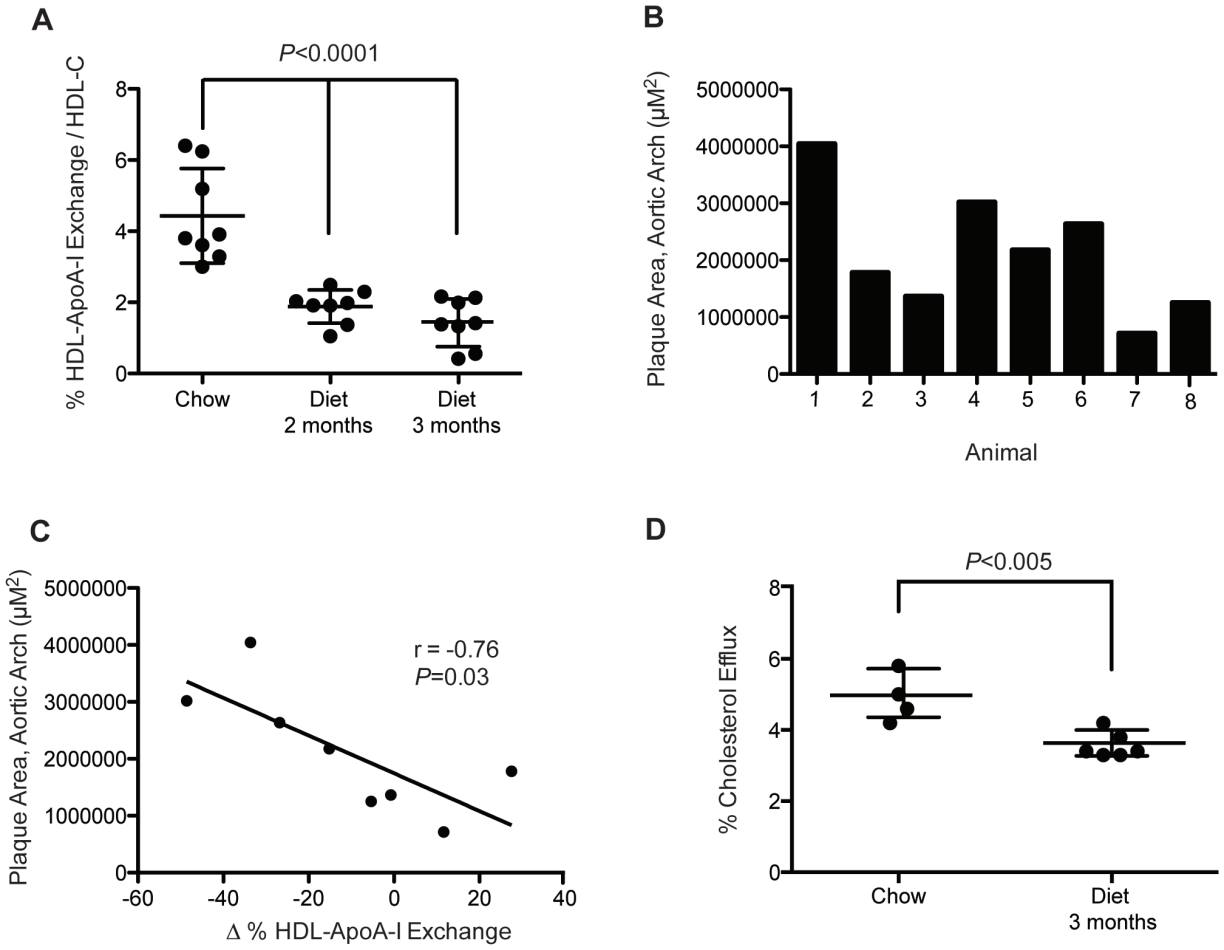
Progression of atherosclerosis is associated with reduced HAE in rabbits. [**A**] HDL-ApoA-I exchange, normalized to HDL-C in rabbits fed normal rabbit chow, then switched to a high fat, high cholesterol diet (0.3% cholesterol, 4.7% coconut oil). Blood was collected at 2 months and 3 months following onset of the diet. HDL was isolated by ultracentrifugation from plasma and HAE was normalized to HDL-C concentration. *P*<0.0001, one-way ANOVA. [**B**] Atherosclerotic plaque area, as measured by histology analysis in the aortic arch. [**C**] Relationship between the change in HAE over months 2 and 3 on the high fat diet and the size of atherosclerotic plaque in the aortic arch. Correlation was determined by linear regression analysis using Pearson's correlation coefficient. [**D**] Total serum efflux capacity from J774 macrophage cells in rabbits on chow diet and after 3 months on the high fat, high cholesterol diet (*P* = 0.003). Statistical significance was determined by two-tailed Student's *t*-test.

HAE of purified HDL was assessed by EPR and normalized to HDL-C to control for differences in HDL induced by diet modification. Significant impairment of HAE was observed after 2 months on the high fat, high cholesterol diet with additional loss of exchange - by 3 months (Figure 4A, *P*<0.0001, one-way ANOVA). Histology analysis of rabbit aortas revealed that the majority of plaque burden was in the aortic arch with significant variable lesional levels from animal to animal (Figure 4B). The degree of change in HAE between months 2 and 3 on diet correlated inversely with plaque burden in this region (Figure 4C, Pearson's r = −0.76; *P* = 0.03).

We examined the effect of a high fat, high cholesterol diet on the capacity of rabbit blood serum to efflux cholesterol from J774 macrophage cells. There was a significant decline in serum HDL efflux capacity after 3 months on the diet compared to serum HDL from the same animals prior to going on the diet (Figure 4D). This is in agreement with earlier studies in humans and monkeys that show an inverse relationship between cholesterol efflux capacity and atherosclerosis [54], [63].

### HDL-ApoA-I Exchange is Impaired in Subjects with ACS

**Figure 5:**
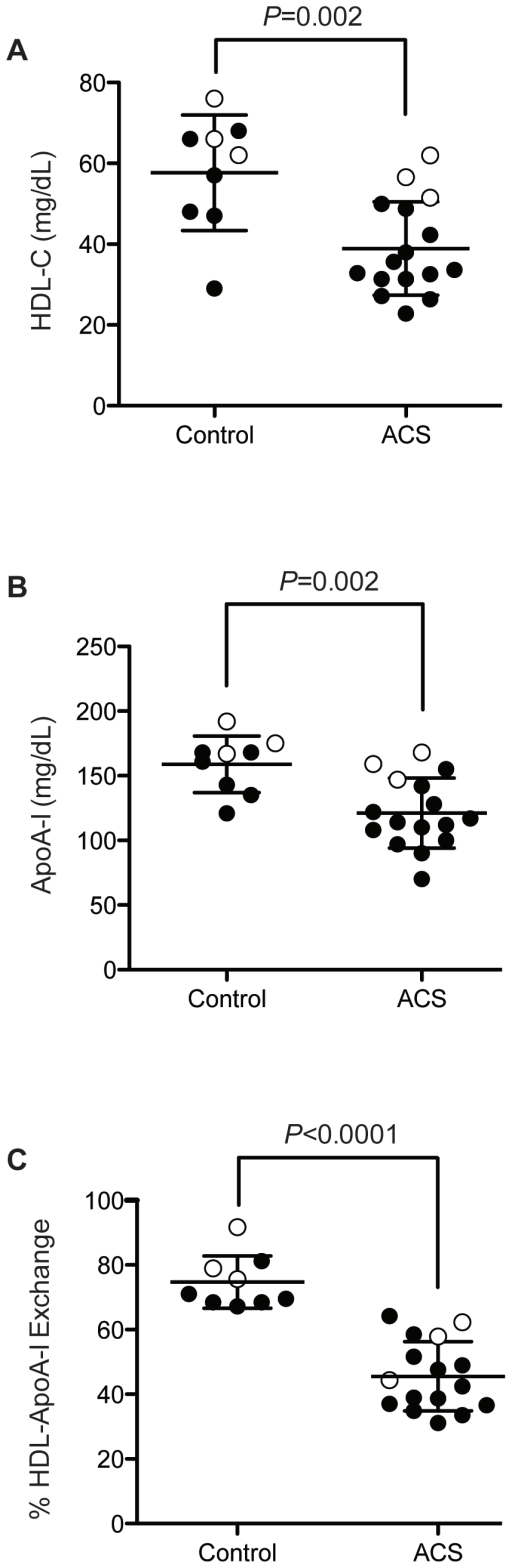
HDL-C, ApoA-I and HDL-ApoA-I exchange (HAE) measured in control and ACS subjects. [**A**] HDL-C and [**B**] apoA-I levels in ACS and control subjects. Significant differences (*P* = 0.002) were observed between the two groups. [**C**] HAE measured in apoB-depleted plasma in control and ACS subjects. Closed circles are males and open circles are females. Statistical significance for [A–C] was determined by performing a two-tailed Student's *t*-tests.

To determine if HAE activity is affected in subjects with known atherosclerosis, we evaluated blood plasma HAE of 16 subjects who experienced acute coronary syndrome (ACS) 3 months prior to plasma collection. Additionally, 9 healthy subjects with no history of coronary artery disease were examined. ACS subjects exhibited significantly lower HDL-C and apoA-I values compared to healthy control subjects (Figure 5A;B and Table 1). EPR analysis was performed on apoB-depleted plasma to maximize scan-to-scan consistency. ACS subjects had significantly lower HAE compared to the control group (*P*<0.0001; Figure 5C and Table 1). Interestingly, there was complete distinction between ACS subjects' HAE values and healthy controls.

**Table 1:** Clinical characteristics of study subjects.

Parameter	Control (n = 9)	ACS (n = 16)
**Age (years)**	35±13*	59±10
**Male (%)**	6 (67%)	13 (81%)
**HDL-C (mg/dL)**	57.7±14.3	38.9±11.6
**ApoA-I (mg/dL)**	158.9±21.9	121.2±27.1
**% HDL-ApoA-I Exchange**	74.7±8.1	45.6±10.7

* Mean reported ± SD.

**Figure 6:**
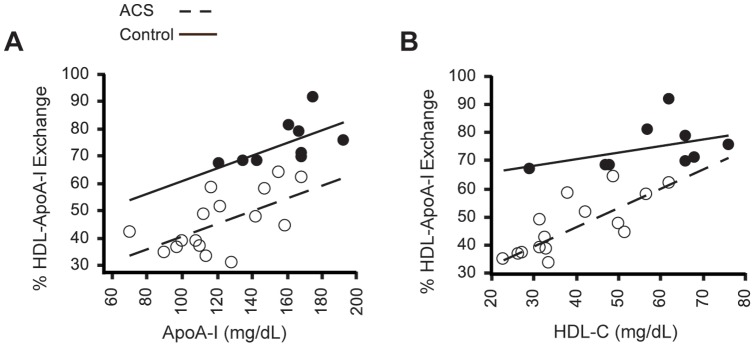
Analysis of HAE in ACS and control subjects with respect to apoA-I and HDL-C levels. [**A**] Analysis of HAE with respect to apoA-I for ACS (open circles) and control subjects (closed circles). HAE is highly correlated to apoA-I (r = 0.89; *P*<0.0001) and increases by 0.23±0.07% for every 1 mg/dL increase in apoA-I in both ACS and control subjects (*P* = 0.002). HAE of ACS subjects is on average 20±4% lower than control patients with the same apoA-I concentration. [**B**] Analysis of HAE with respect to HDL-C shows that HAE is also highly correlated to HDL-C (r = 0.90; *P*<0.0001). For ACS patients, HAE increases by 0.69±0.17% for every 1 mg/dL increase in HDL-C and 0.23±0.19% for control patients (*P* = 0.0006), but the difference between the slopes is not significant (*P* = 0.09). Statistical significance was determined by two-way ANOVA, and post hoc comparisons by custom tests of parameters. Interaction p-values greater than 0.20 were dropped in favor of pooled estimates.

To investigate the relationship between HAE, HDL-C and apoA-I levels, we performed regression analyses of the ACS and control groups using a two-way ANOVA to compare the two groups (Figure 6). HAE was highly correlated with apoA-I levels (Figure 6A, r^2^ = 0.89; *P*<0.0001). Regardless of ACS status, HAE increased by 0.23±0.07% for every 1 mg/dL increase in apoA-I (*P* = 0.002). However, the HAE of ACS subjects was 20±4% lower than controls having the same apoA-I concentration, an observation consistent with a qualitative deficiency in the apoA-I of ACS subjects. There was also a strong correlation between HAE and HDL-C levels (Figure 6B, r = 0.90; *P*<0.0001), but the differences in HAE existed primarily between the subjects with low HDL-C. Control subject's HAE appeared independent of HDL-C, increasing 0.23±0.19% for every 1 mg/dL increase in HDL-C (*P*>0.20). However, among ACS subjects, HAE increased by 0.69±0.17% per 1 mg/dL HDL-C (*P* = 0.0006). Thus, at low HDL-C concentrations (∼30 mg/dL), the difference between control and ACS HAE is −29±6% (*P* = 0.0002) while at higher HDL-C concentrations (∼60 mg/dL), the difference is −14±5% (*P* = 0.01).

### HDL-ApoA-I Exchange Correlates with ABCA1-mediated Cholesterol Efflux Capacity

**Figure 7:**
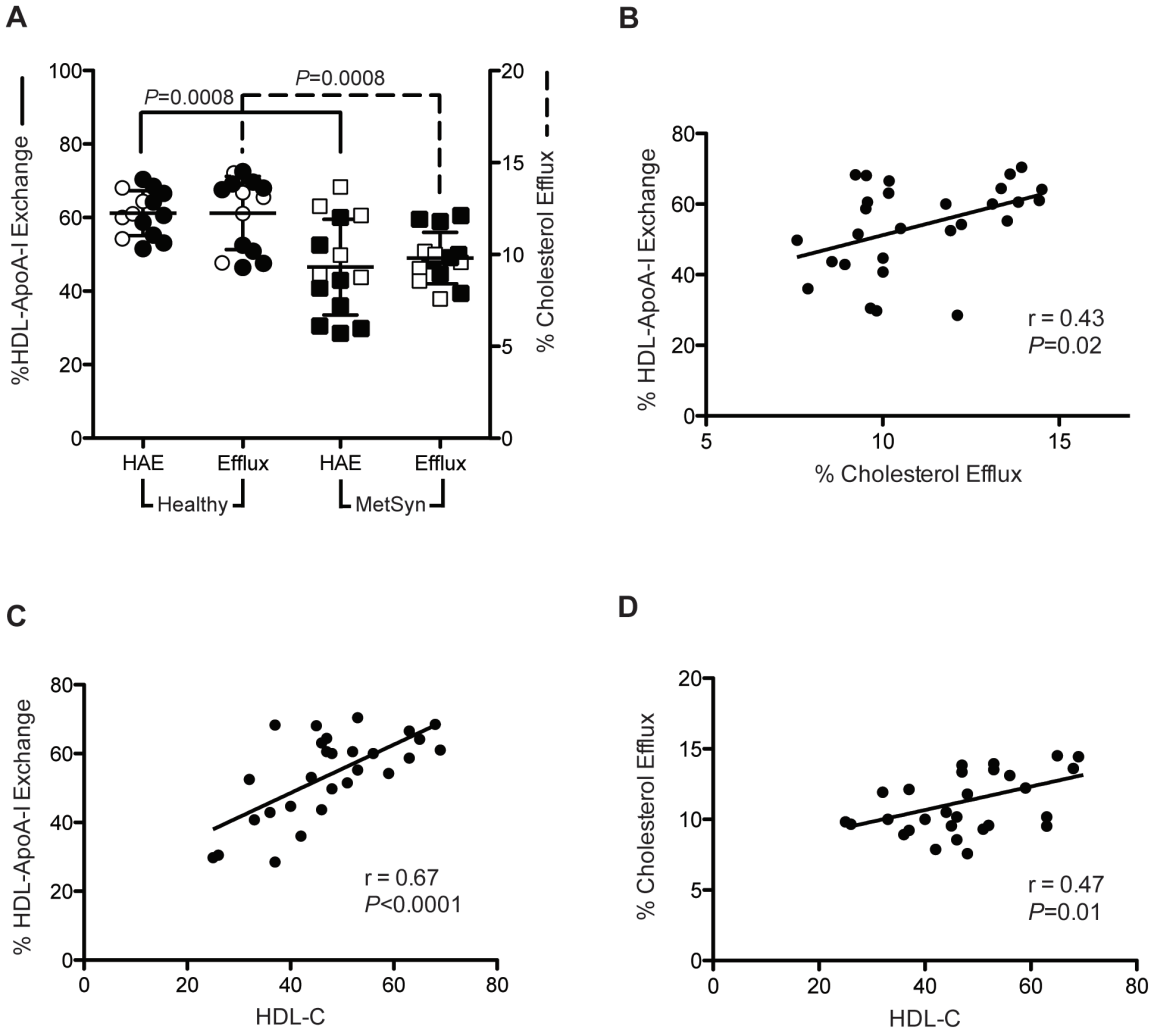
Comparison of HDL-ApoA-I exchange to ABCA1-mediated cholesterol efflux capacity in subjects with metabolic syndrome. [**A**] In-plasma HAE is compared to ABCA1-mediated cholesterol efflux capacity in BHK cell expressing mifepristone-inducible human ABCA1 for subjects with metabolic syndrome (squares) and healthy controls (circles). Black circles or squares are males and open circles or squares are females. [**B**] Correlation of ABCA1-mediated cholesterol efflux and HAE, [**C**] HDL-C and HAE, and [**D**] HDL-C and ABCA1-mediated cholesterol efflux. Correlation was determined by linear regression analysis using Pearson's correlation coefficient.

We compared HAE and ABCA1-mediated cholesterol efflux capacity in subjects diagnosed with metabolic syndrome and a corresponding healthy control group. Since ABCA1-mediated efflux is dependent upon the availability of lipid-poor apoA-I cholesterol recipient [23], cholesterol efflux measurements were performed using BHK cells expressing mifepristone-inducible human ABCA1 [64]. Cholesterol efflux and HAE were significantly lower in subjects with metabolic syndrome compared to healthy control subjects (Figure 7A, *P* = 0.0008 for both, 2-tailed Student's *t*-test). Average cholesterol efflux was 12.3%±0.5 for the control group (n = 14) and 9.8%±0.4 for the metabolic syndrome group (n = 14), while HAE was 61.2%±1.6 and 47.3%±3.3 for the control and metabolic syndrome subjects, respectively.

HAE was impaired in metabolic syndrome patients similar to ABCA1-mediated cholesterol efflux (Figure 7B; Pearson's r = 0.43; *P* = 0.02). This suggests that the displacement/desorption rate of apoA-I from HDL, as measured by HAE, can also serve as an indicator of cholesterol efflux capacity. Both HAE and ABCA1-mediated efflux correlated significantly with HDL-C (Figure 7C;D *P*>0.0001 and *P* = 0.01, respectively).

## Discussion

While plasma HDL-C is clearly associated with CAD in longitudinal population studies, HDL-C is not, on an individual basis, a good predictor of a patient's predisposition for CAD. Furthermore, both the Framingham Offspring study and the MESA study found that nearly 40% of CAD patients had normal or elevated HDL-C levels [65], [66]. Similarly, in the IDEAL trial, the highest risk estimates were seen in patients with HDL-C levels above 70 mg/dL [67], [68]. These studies suggest that there may be a dysfunctional pool of HDL that can lead to abnormally high HDL-C and/or CAD. Because HDL-C measures include both healthy and dysfunctional HDL particles, HDL-C levels do not accurately predict CAD risk at the individual level, underscoring the importance of HDL function in assessing CAD risk.

The laboratories of Drs. Rader and Rothblat recently demonstrated that the ability of human plasma HDL to promote cholesterol efflux from cultured macrophages varies significantly among individual subjects, despite similar levels of HDL-C and apoA-I [53]. They further determined that the sterol efflux capacity of plasma HDL strongly associates with CAD status, independent of HDL-C [54]. This metric of HDL function exhibited a greater inverse correlation odds ratio (0.75; 95% CI) than HDL-C (0.85; 95% CI) and is a more accurate predictor of CAD than HDL-C with *P*<0.002 versus *P*<0.09, respectively.

While very promising, measuring HDL sterol efflux capacity of human plasma is a laborious, costly, requires radioactivity and cultured cells, and is difficult to standardize, thus precluding broad clinical application. We have capitalized on the fact that the availability of lipid-poor apoA-I is an essential component to the ABCA1-mediated mobilization of cellular cholesterol and excess lipids to create a rapid, robust and scalable assay to quantify a cholesterol efflux-relevant aspect of HDL function [25]. Because apoA-I is synthesized in the liver and transported to the periphery on HDL particles, the conformational adaptability of apoA-I, and thus its ability to exchange off HDL particles [23], [69], [70], is important for substrate generation in the intima. Thus, HAE provides a measure of apoA-I's ability to desorb from HDL, a rate-limiting process that precedes ABCA1-mediated cholesterol efflux.

Despite measuring a different step of reverse cholesterol transport, our findings indicate that HAE and cell-based ABCA1-mediated cholesterol efflux yield similar insight into HDL function. In subjects with metabolic syndrome, HAE and ABCA1-mediated efflux were reduced to a similar degree compared to the control group (Figure 7A), and there were strong correlations between cholesterol efflux capacity and HAE (Figure 7B).

In an animal model of diet-induced atherosclerosis, we observed substantial declines in HAE after rabbits were transitioned from chow to a high fat, high cholesterol diet. Examination of the aortas of individual animals revealed varying degrees of atherosclerotic plaque and the highest aortic plaque burden was associated with the largest negative changes in HAE between months 2 and 3 on the diet (r = −0.76, *P* = 0.03; Figure 4C). Notably, when HAE was normalized to HDL-C, the degree of HAE per HDL was dramatically reduced, suggesting that HDL became less functional over the course of the diet. This was confirmed by cell-based cholesterol efflux assays, wherein rabbit HDL cholesterol efflux capacity was impaired for the animals after 3 months on a high fat, high cholesterol diet (Figure 4D). Together, these observations support the hypothesis that loss of HAE is associated with atherogenesis.

Although confounded by factors such as age, diabetic status, hypertension, and obesity, indicators of chronic inflammation (C-reactive protein, fibrinogen, white-cell count, and platelet activating factor acetyl hydrolase) significantly associate with increased risk of CAD [71]–[74]. Chronic inflammation involves the activation of macrophages, which can produce an oxidative environment in the artery intima due largely to the production of MPO [52], [75], [76]. This can lead to chemical modification of HDL [62], wherein apoA-I is the primary target [40], [43]. Plasma levels of MPO and MPO-induced posttranslational modification are positively associated with CAD, and elevated MPO activity is observed in atherosclerotic lesions [40], [77], [78]. MPO-derived oxidative modification of apoA-I impairs ABCA1-mediated cholesterol mobilization [29], [43]–[45]. Similarly, the HAE of isolated human HDL treated with MPO was significantly impaired in a dose-dependent fashion, relative to levels of H_2_O_2_ and MPO, demonstrating that the EPR-based assay is sufficiently sensitive to detect changes in HDL function elicited by MPO (Figure 3).

Analysis of HAE in human plasma revealed a significant difference between healthy control subjects and patients with either ACS or metabolic syndrome, demonstrating that the EPR-based HAE assay can detect dysfunctional HDL in subjects both prior to and following a cardiovascular event. When we compared healthy control and ACS subjects with similar HDL-C and apoA-I levels, the ACS subjects all had significantly lower HAE (Figure 5C), consistent with the conclusion that HAE is a functional parameter of HDL independent of HDL-C and apoA-I. Importantly, when we analyzed HAE with respect to apoA-I and HDL-C levels for healthy and ACS subjects, the ACS subjects exhibited consistently lower HAE at comparable concentrations of apoA-I or HDL-C (Figure 6). This was particularly true for apoA-I, where for any given apoA-I concentration HAE was ∼20% lower for ACS subjects compared to the control group (Fig. 6A). This links a qualitative deficiency in apoA-I to CAD. These data, along with the finding that MPO-induced oxidative modification impairs HAE *in vitro* (Figure 3 and [25]), are strong evidence that reduced HAE is a biomarker of cardiovascular disease. Furthermore, our findings support the notion that HDL function (cholesterol efflux capacity, HAE) is, on an individual basis, more indicative of cardiovascular health than HDL-C alone [1], [5], [54], [79]. Ongoing outcome trials of HDL-modifying agents will provide specific cohorts of patients to determine the validity of this biomarker of HDL function in determining outcomes.

HDL dysfunction likely appears long before a diagnosis of cardiovascular disease. Patients with metabolic syndrome, for example, have decreased HDL-C and increased blood glucose levels, and are at greater risk for CAD-related mortality [80]. In selecting the subjects with metabolic syndrome, individuals with diagnosed cardiovascular disease were specifically excluded [48]. Analysis of HAE and efflux capacity in subjects with metabolic syndrome revealed that HDL dysfunction is detectable prior to a diagnosis of CAD (Figure 7A). In light of our findings that HDL function declines with the progression of atherosclerosis, these results indicate CAD-related mechanisms are already present in these patients. As elevated blood glucose is one of the hallmarks of metabolic syndrome, a potential mechanism of HDL dysfunction is non-enzymatic glycation of apoA-I. Recently, a pattern of atherogenesis has been observed in type 2 diabetics, where the degree of non-enzymatic apoA-I glycation correlated with the progression of atherosclerosis [28]. Therefore, a possible future clinical application of HAE may be to monitor HDL function in patients with metabolic syndrome or diabetes, where declining HAE would indicate progression of pathology and risk/status of atherosclerosis and CAD.

Measurement of HAE adds an important level of detail to existing blood lipid analyses, directly reporting the functional status of HDL via assessment of the conformational plasticity of apoA-I. It may prove especially useful in patients with higher HDL-C levels where developing CAD might be otherwise unsuspected. For example, HAE analysis could benefit women, who typically have higher HDL-C levels compared to men, making determination of cardiovascular risk more difficult [81]. This will be an important focus in future studies.

Our findings pave the way for new investigations that combine measures of HDL function that include HAE, mass spectrometry to investigate apoA-I posttranslational modification, and ABCA1-mediated cholesterol efflux, to gain a deeper understanding of the molecular underpinnings of cardiovascular disease. Because of its scalability, precision, and modest sample requirements, we envision that the HAE assay could be readily translated to the clinic wherein it would provide a rapid and precise assessment of a patient's HDL functional status and thereby a more accurate determination of CAD risk/status.
